# Are characteristics associated with strong Black womanhood linked to depression in older Black women?

**DOI:** 10.1177/17455057241274923

**Published:** 2024-09-05

**Authors:** Christy L Erving, Cleothia Frazier, KJ Davidson-Turner

**Affiliations:** 1 Department of Sociology, Population Research Center, The University of Texas at Austin, Austin, TX, USA; 2 Department of Sociology and Criminology, Social Science Research Institute, The Pennsylvania State University, University Park, PA, USA

**Keywords:** Black women, older adults, depression, strong Black woman, superwoman schema

## Abstract

**Background:**

Older Black women experience structural and intersectional disadvantages at the intersection of age, race, and gender. Their disadvantaged social statuses can translate into serious psychological health consequences. One concept that may aid in understanding psychosocial determinants of older Black women’s depression risk is the “Strong Black Woman,” which suggests that Black women have supernatural strength amidst experiencing adversity and are expected to “be strong” for others by providing self-sacrificial aid without complaint.

**Objectives:**

Drawing inspiration from the “Strong Black Woman” concept, the current study examined whether three psychosocial factors (i.e., mastery, anger suppression, and relational demands (from spouse, children, relatives, and friends)) were associated with depressive symptoms, clinically significant depressive symptoms, and lifetime professionally diagnosed depression among older Black women (i.e., ages 50 years and older).

**Design:**

This was a cross-sectional study. Data were drawn from the 2010 to 2012 waves of the Health and Retirement Study (*N* = 1,217).

**Methods:**

For past-week depressive symptoms, ordinary least squares regression analyses were conducted, and beta coefficients were reported. For clinically significant depressive symptoms (i.e., reporting three or more depressive symptoms in the past week) and lifetime professionally diagnosed depression, binary logistic regression analyses were performed, and odds ratios were reported.

**Results:**

Higher levels of mastery were associated with lower risk for depressive symptoms and depression. Anger suppression was associated with higher risk for depressive symptoms and depression. Demands from children and one’s spouse were associated with higher depressive symptoms while demands from family were associated with risk for lifetime depression diagnosis. Not having a spouse was associated with heightened risk of depressive symptoms and depression. Interestingly, demands from friends were not associated with depressive symptoms nor diagnosed depression.

**Conclusion:**

Study findings revealed important nuances in the determinants of depression among older Black women which, in turn, has implications for research and mental health care provision in this population.

## Introduction

Despite often being pillars within their communities, families, and workplaces, older Black women experience depression and/or depressive symptoms at alarming rates. One study, for instance, reported, that 20% of Black women 50 years and older “felt depressed” in the past year.^1^ Relative to their White and Black male counterparts, Black women also report experiencing higher depressive symptoms.^2,3^ Even when not meeting criteria for a depression diagnosis, subthreshold depression among older Black women is more severe compared to White women.^4^ Moreover, older Black women are more commonly underdiagnosed with depression relative to older White women.^4^ Relatedly, misdiagnosis of depression (e.g., with a diagnosis of a conduct or psychotic disorder instead) remains a prominent diagnostic concern for Black Americans in general and Black women in particular.^5^

Given that one out of five Black women have experienced depression in their lifetime,^1^ it is imperative to understand the specific determinants of depression within this population. Research indicates that physical pain,^6^ poor self-rated physical health,^1^ and multiple chronic conditions^7^ are critical determinants of older Black women’s depressive symptoms. In addition, social factors such as stressful life events, material hardship, discrimination, stress exposure,^8^ social support,^1^ and romantic relationship quality^9^ are also linked to depression in older Black women. The current study adds to a growing body of work that examines the interplay between social factors and depression/depressive symptoms among older Black women. Specifically, we investigate the extent to which three characteristics (anger suppression, relational demands, and mastery) associated with the concept of “Strong Black Womanhood” are related to depressive symptomatology and depression diagnosis among older Black women. In addition to the concerning epidemiological patterns described above, there are also theoretical reasons to focus on psychosocial factors related to depression in older Black women. Here, we draw on the intersectionality and the superwoman schema (SWS) frameworks to investigate three potential drivers of older Black women’s risk for depression.

### Theoretical framework

The intersectionality framework argues that multiple systems of stratification intersect to produce unique inequalities for members of socially disadvantaged groups^10,11^ which, in turn, produce health advantages for some groups and health disadvantages for others.^12,13^ The distinction between inter-categorical and intra-categorical analytic approaches to intersectionality research is apropos here.^14^ Inter-categorical intersectional studies are concerned with comparing socially meaningful categories of people or groups (e.g., women compared to men; Black Americans compared to Asian Americans), and much of quantitative health scholarship within the social sciences has taken this approach (see Harari and Lee^15^ for a review). Intra-categorical complexity, however, is revealed in intersectional studies focused on a single group experiencing multiple disadvantaged social statuses (e.g., Latina lesbian women). Though inter-categorical (i.e., comparative studies) studies are needed to assess the magnitude of health differences between different status groups (i.e., a descriptive intersectionality^16^), intra-categorical studies in health research have the potential to identify social determinants specific to the group under study (i.e., an analytic intersectionality^16^). This study adopts an intra-categorical approach to unveil the specific psychosocial correlates of depression among older Black women.

Integrating ideas related to the mythical “Strong Black Woman” (SBW) notion,^17–19^ the SWS draws from Black feminist thought and intersectionality scholarship to conceptualize the ways Black women are socialized to selflessly care for others at their own expense while maintaining a façade of strength.^20–22^ In developing the framework, Woods-Giscombe and colleagues^22^ identified five distinct, but related dimensions of SWS: obligation to present an image of strength, obligation to suppress emotions, resistance to vulnerability, intense motivation to succeed despite limited resources, and an obligation to help others at one’s own expense. SWS endorsement is associated with higher risk of depressive symptoms among Black women in emerging adulthood^23^ and in early mid-life.^24^ Nevertheless, research suggests that SWS could be especially salient for older Black women who have faced life course structural adversities (e.g., de jure and de facto segregation in the United States) yet have survived to mid- or later-life^25^ (see Platt and Fanning^26^ for an exception).

Despite findings suggesting that SWS can be psychologically maladaptive, SWS may also be cast as a protective mechanism that has enabled older Black women to cope with structural inequalities and the interpersonal gendered racism they have navigated across the life course. Baker and colleagues^25^ characterized strong Black womanhood as an indicator of “successful aging” among Black women, noting that independence and perseverance may serve as resilience mechanisms. Relatedly, some scholars recommend taking a “strengths-based” therapeutic approach for mental health practitioners working with Black women.^27^ This perspective presents an alternative interpretation of the SWS which could possibly operate as an adaptive orientation to life that reduces depression risk and depressive symptoms among older Black women. As of this writing, no study has examined the effects of SWS on older Black women’s depression or depressive symptoms.

In the current study, inspired by the SWS framework, we focus on three related psychosocial factors: *mastery* (related to SWS dimensions of strength and motivation to succeed), *anger suppression* (related to SWS dimension of emotion suppression), and *relational demands* (related to SWS dimension of obligation to help others).

Below, we review the literature examining these factors as they relate to depressive symptoms, and (when available) draw on literature focused on Black women. Though these psychosocial factors do not perfectly align with the SWS dimensions, the current study can motivate future research that assesses the mental health impact of SWS endorsement on older Black women.

### Mastery

Mastery refers to the “extent to which one regards one’s life-chances as being under one’s own control in contrast to being fatalistically ruled” (p. 5).^28^ Mastery acts as an important mental health-protective resource among older adults. Despite evidence that mastery may decline at later ages,^29,30^ older adults who maintain a powerful sense of mastery demonstrate an ability to cope with acute and chronic stress, deal with life challenges, and engage in protective health behaviors.^30,31^ Studies show that in older adults, mastery is associated with lower levels of psychological distress,^32^ depression,^33^ and depressive symptoms.^34^ Among Black women, a high sense of mastery is inversely associated with psychological distress^35^ and is protective against depressive symptoms.^36^

However, older Black women who may have faced a lifetime of individual and institutional stressors may be more susceptible to poor mental health in late life due to diminished mastery.^37^ Furthermore, exposure to chronic stress (e.g., from SWS) can diminish mastery and increase vulnerability to poor psychological health.^37,38^ According to Thomas and González-Prendes,^39^ while the notion of Strong Black Woman is associated with strength, there is a paradox for Black women, who may simultaneously feel a sense of powerlessness to change the external circumstances that require the need to self-sacrifice and care for others. Thus, this sense of powerlessness may indicate a lack of control (i.e., lower mastery), which could negatively impact older Black women’s mental health. However, it is important to note that for older Black women who adopt SWS, an elevated level of mastery may be indicative of psychological resilience against the need to show strength, demonstrating an ability to manage chronic stress.

### Anger suppression

Despite evidence that anger is an emotion that declines with age,^40^ older adults may still experience anger when faced with changing health status and social relations in later life. According to Spielberger et al.,^41^ anger is multidimensional and is comprised of internalized anger, externalized anger, and anger control. Internalized anger, or anger suppression, refers to the frequency with which angry feelings are experienced but not expressed.^41^ A central feature of psychological disorders is emotion dysregulation^42,43^ and anger suppression, which is a maladaptive way of expressing anger, is associated with psychological costs and maladjustment.^44^ Often assessed with the State-Trait Anger Expression Inventory (STAXI), research shows that anger suppression is associated with both depression and depressive symptoms.^44–46^ Understanding the association between anger suppression and depression is important because research shows that anger is associated with greater depression severity, increased suicide risk, poorer interpersonal relationships, and poor health outcomes.^47^ Moreover, there is evidence that depressed people engage in more anger suppression due to fear of the consequences associated with outward expressions of anger.^48^ Anger suppression may be especially concerning for older Black women who adopt the notion of “Strong Black Woman.”

Anger suppression among older Black women who adopt the SBW persona represents a form of “self-silencing.”^49^ Self-silencing reflects behavior that inhibits self-expression to maintain relationships and to circumvent retaliation, loss, and conflict.^50^ Refusal to express anger can have serious mental health consequences over time^51^ and may lead to subsequent depression and depressive symptoms.^52^ In fact, self-silencing is not only a hallmark characteristic of SBW, but Abrams and colleagues^49^ also found that the more Black women felt obligated to display strength, the more likely they were to silence themselves and, in turn, develop depressive symptoms. The unrealistic demands and problematic expectations of stoicism leave Black women with feelings of frustration and anger that cannot be expressed because they do not align with the image of strength.^53^ As noted by González-Prendes and Thomas,^53^ the combination of powerlessness and strength is a paradox for Black women. The emotional outcome of this paradox is the experience of frustration and anger, which Black women often do not express. In addition, Black women suppress anger to avoid negative stereotypes associated with being “unfeminine,” the “Angry Black Woman,” or being viewed as “aggressive.”^54^ As such, the inability to openly express anger may increase older Black women’s vulnerability to depression and depressive symptoms.

### Relational demands

Scholars have turned to protective factors including close relationships and relationship quality to better understand the ways Black women leverage their social networks to retain positive mental health. For instance, Baldwin-Clark and colleagues^1^ found that being married was associated with lower risk of past-year and lifetime depression. Drawing on a sample of Black women living in Georgia and Iowa, Hanus and colleagues^9^ provided additional context by demonstrating that marriage alone is not mental health protective; instead, having a romantic partnership (i.e., married, cohabiting, or exclusively dating) *and* having high relationship quality (e.g., relationship satisfaction, partner warmth) was linked to lower depressive symptoms.

When expanding to other network members, Black women appear to experience *higher* depressive symptoms when accessing tangible support from friends and relatives.^3^ This finding runs counter to the broader literature which suggests that receipt of social support is beneficial to mental health.^55,56^ Since one characteristic of SWS involves obligation to help others to the point of neglecting self-care, we build on this scholarship to examine the “dark side” of relationships^57^ to assess whether relational demands are associated with depression/depressive symptomatology in Black women. We also recognize that the social ecology of Black women’s lives involves not only their spousal relationship, but also often entails an extensive network of relatives, friends, and children. Thus, we ascertain how relational demands within diverse types of relationships are associated with risk for depression.

## Methods

Our analysis used a cross-sectional study design drawing data from the Health and Retirement Study (HRS), a nationally representative, longitudinal study. We restricted our analysis to the 2010 and 2012 waves obtained from the RAND HRS 2020 Longitudinal File. The RAND HRS longitudinal file was merged with the 2010 and 2012 Psychosocial and Lifestyle Questionnaire (leave behind survey (LBS)).^58^ The LBS was first introduced in 2006, with half samples receiving the option to complete the LBS every 2 years. We used data from the 2010 and 2012 waves because these were the most recent waves that included all measures of interest for the study (i.e., perceived mastery, anger suppression, and relational demands).

In total, the combined 2010 and 2012 LBS contained 15,717 respondents. In the HRS, respondents were queried about their race/ethnicity using the following question: “What race do you consider yourself to be: White, Black or African American, American Indian, Alaska Native, Asian, Native Hawaiian, Pacific Islander, or something else?” For this analysis, we only included respondents who identified as “Black or African American” and reported their sex as female, which restricted our sample to 1,645 adults. We also restricted our sample to those who were 50 years of age and older, which reduced our sample to 1,578 respondents. We performed listwise deletion for any missing values; therefore, we excluded 28 respondents with missing data for the mastery scale, 37 respondents with missing data for anger suppression, 17 respondents with missing data for spousal demands, 77 respondents with missing data for child demands, 50 respondents with missing data for family demands, and 140 respondents with missing data for friend demands. In addition, 10 respondents were dropped for missing depressive symptoms and 2 respondents were dropped for missing doctor-diagnosed depression. After dropping missing cases, the analytic sample included 1,217 Black women. A power analysis was not conducted because we used all available data from the HRS public use file for inclusion in our analytic sample (i.e., respondents who identified as a Black woman and were aged 50 years and older).

### Dependent measures

Given the multidimensionality of depression and challenges with depression under- and misdiagnosis among Black women,^59^ we include three measures in our analysis: (1) depressive symptoms in the past week, (2) “clinically significant” depressive symptoms in the past week, and (3) a lifetime indicator of professionally diagnosed depression.

First, *past-week depressive symptoms* were assessed using the 8-item version of the Center for Epidemiological Studies Depression Scale (CES-D).^60^ We employed the standard abbreviated 8-symptom scale of the CES-D measure available in the HRS RAND (0 = no symptoms, 8 = all eight symptoms). Respondents were asked to respond to whether they experienced in the past week any of the following (0 = no, 1 = yes): (1) I felt depressed; (2) I felt everything I did was an effort; (3) My sleep was restless; (4) I was happy; (5) I felt lonely; (6) I enjoyed life; (7) I felt sad; and (8) I could not “get going.” The responses to the eight questions were then combined to create a total depressive symptom score that ranged from 0 (no symptoms) to 8 (all symptoms). Second, we developed a binary measure of depressive symptoms: based upon prior work that has created measures of depression based on clinical cutoffs,^61^ we created a measure of *clinically significant past-week depressive symptoms*. Respondents who reported less than 3 CES-D symptoms were categorized together (<3 symptoms) and those who reported having three or more depressive symptoms were grouped together (3+ symptoms) and defined as having clinically significant depressive symptoms. Third, in addition to the CES-D scale questions, respondents were also asked whether their doctor ever provided them with a depression diagnosis. We created *lifetime diagnosed depression* using a dichotomous depression variable based on the self-report of whether a doctor ever diagnosed the respondent with depression (0 = no, 1 = yes).

### Independent measures

#### Mastery (related to SWS dimensions of strength and motivation to succeed)

Perceived mastery was based on a 5-item version of the perceived mastery scale.^62,63^ Respondents were asked to indicate how much they identified with each of the five items: (1) I can do just about anything I really set my mind to; (2) When I really want to do something, I usually find a way to succeed at it; (3) Whether or not I am able to get what I want is in my own hands; (4) What happens to me in the future mostly depends on me; and (5) I can do the things that I want to do. Response options included: 1 = strongly disagree; 2 = somewhat disagree; 3 = slightly disagree; 4 = slightly agree; 5 = somewhat agree; or 6 = strongly agree. We then averaged the scores from the five mastery statements to create an index of mastery (1–6).

#### Anger suppression (related to SWS dimension of emotion suppression)

Our measure of anger suppression was created based on the STAXI measures included in the HRS.^64^ We created our measure based on the trait anger anger-in scale (i.e., anger suppression) dimension included in the scale that captures whether respondents were more likely to respond to various situations angrily. The anger suppression measure was based on respondents’ answers to four statements: (1) When I am feeling angry or mad, I keep things in; (2) When I am feeling angry or mad, I withdraw from people; (3) When I am feeling angry or mad, I am irritated more than people are aware; (4) When I am feeling angry or mad, I am angrier than I am willing to admit. Response categories included: 1 = almost never; 2 = sometimes; 3 = often; or 4 = almost always. We then averaged the anger-in statements to create an average anger suppression score.

#### Relational demands (related to SWS dimension of obligation to help others)

Perceived relational demands were created based on the scales included in the HRS to assess the quality of the social relationships of respondents.^65,66^ From the perceived negative social support items, we relied on the question: “How often do they make too many demands on you?” Respondents were asked to indicate how they related to each statement: 1 = a lot; 2 = some; 3 = a little; or 4 = not at all. Four sources of relational demands were assessed: spouse, child, family, and friend. Of note, for spousal and children demands, we wanted to capture respondents who were not married and who did not have children. Thus, we operationalized those specific sources of demands as follows: for *spousal demands*, respondents were assigned to one of three categories: little/no spousal demands (reference), some/a lot of spousal demands, or no spouse. To distinguish between those who had a spouse and those who did not, those who were divorced/separated, widowed, and never married were assigned to the “no spouse” group. Those who were married were attributed to one of the two spousal demand categories. Regarding the “no spouse” category, it is noteworthy that 776 respondents (64%) did not have a spouse. Specifically, 28% of respondents were separated or divorced, 24% were widowed, and 12% were never married. We conducted supplemental analysis to assess whether there were distinctions in depression across separated/divorced, widowed, and never-married respondents compared to those who were married. Overall, irrespective of their specific status, “no spouse” respondents experienced higher risk of depressive symptoms and/or depression compared to those who were married. Thus, we opted to retain the “no spouse” broad category.

For *child demands*, respondents were assigned to one of three categories: little/no child demands (reference), some/a lot of child demands, or no child. For family and friend demands, there was no option for respondents to report “no” family or friends. Thus, we created binary measures of family and friend demands. For *family demands*, respondents were assigned to one of two categories: little/no family demands (reference) or some/a lot of family demands. For *friend demands*, respondents were assigned to one of two categories: little/no friend demands (reference) or some/a lot of friend demands.

### Controls

Consistent with prior literature, we adjusted all models for several controls. *Age* was self-reported at the time of the interview in either 2010 or 2012. We created a categorical variable indicating *education attainment*: (1) less than high school (reference); (2) General Education Diploma (GED)/high school; (3) Some college; and (4) college graduate. *Household income* was based on the self-reported household income measure that includes the income of the respondent and the spouse, if applicable. Income was logged and retained as a continuous variable. *Labor Force Status* was assessed based on four categories: full-time employment (reference), part-time employment, unemployed, or retired. Those who reported being unemployed, disabled, or not in the labor force were all assigned to the “unemployed” category. *Physical conditions* were measured by creating a scale of conditions from 0 to 5. Respondents who had five or more health conditions were assigned to 5. The reported conditions included arthritis, high blood pressure, diabetes, cancer, lung disease, stroke, and heart disease.

### Statistical analysis

To examine the impact of mastery, anger suppression, and relational demands on depression using past-week depressive symptoms, we estimated ordinary least squares regression models for each domain, and results were reported as unstandardized beta coefficients. We performed binary logistic models to estimate different impacts of clinically significant past-week depression and lifetime diagnosed depression for mastery, anger suppression, and relational demands. Results were displayed in odds ratios (OR). All models were adjusted for age, education, household income, labor force status, and physical conditions. We evaluated statistical significance at an α-level of 0.05. Model analyses were estimated using STATA 16.1 (Stata Statistical Software: Release 16. 1. 2020; StataCorp LLC, College Station, TX).

## Results

### Sample characteristics

Table 1 provides demographic characteristics of our sample. The average number of past-week depressive symptoms for Black women was 1.80 (standard deviation (SD) = 2.05). Approximately, 73% of Black women had less than three depressive symptoms and 27% had three or more symptoms (i.e., clinically significant depressive symptoms). For doctor-diagnosed depression, nearly 80% of Black women had never been diagnosed with depression while approximately 20% had a professional diagnosis.

**Table 1.** Demographic characteristics of sample (*N* = 1,217).

Measures	Mean or %	SD	Range
Dependent measures			
Depressive symptoms (CES-D)	1.80	2.05	0–8
Clinically significant depressive symptoms			
Less than three symptoms	72.80%		
Three or more symptoms	27.20%		
Lifetime professionally diagnosed depression			
No	79.54%		
Yes	20.46%		
Independent measures			
Mastery	4.79	1.15	1–6
Anger suppression	2.08	0.67	1–4
Spousal demands			
No spouse	63.76%		
Little/no spousal demands (*reference*)	24.40%		
Some/a lot of spousal demands	11.83%		
Child demands			
No child	7.15%		
Little/no child demands (*reference*)	68.94%		
Some/a lot of child demands	23.91%		
Family demands			
Little/no family demands (*reference*)	80.69%		
Some/a lot of family demands	19.31%		
Friend demands			
Little/no friend demands (*reference*)	91.13%		
Some/a lot of friend demands	8.87%		
Controls			
Age (years)	63.68	9.68	50–98
Education			
Less than high school (*reference*)	22.76%		
GED/high school	31.39%		
Some college	29.58%		
College graduate	16.27%		
Household income (logged)	9.93	1.65	0–13.30
Labor force status			
Full-time (*reference*)	27.77%		
Part-time	13.72%		
Unemployed	11.91%		
Retired	46.59%		
Physical conditions (0–5+)	2.13	1.33	0–5

Source: Health and Retirement Study, 2010–2012.

CES-D: Center for Epidemiological Studies Depression Scale; GED: General Education Diploma; SD: standard deviation.

On average, Black women scored high on mastery with an average score of 4.79 (SD = 1.15). The mean anger suppression score was 2.08 (SD = 0.67). Nearly two-thirds of the sample (64%) did not have a spouse, 24% reported little or no spousal demands, and 12% reported some or a lot of spousal demands. Regarding child demands, approximately 69% reported little or no child demands, 24% reported some or a lot of child demands, and 7% did not have children. Most of our sample (81%) reported little or no family demands, with only 19% reporting some or a lot of family demands. Approximately 91% reported having little or no friend demands while 9% reported some or a lot of friend demands.

The mean age of Black women in our sample was approximately 64 years old (SD = 9.68), with the youngest participant being 50 years old and the oldest participant being 98 years old. Approximately 23% did not graduate from high school while 31% obtained a GED or high school degree, 30% had some college experience, and 16% were college graduates. The mean household income was $39,774 (9.93 logged). Given that our sample was representative of an older population, a substantial percentage was retired (47%). Approximately 28% were employed full-time, 14% were employed part-time, and 12% were unemployed at the time of the survey. In addition, Black women had on average 2.13 (SD = 1.33) physical conditions.

### Depressive symptoms

Table 2 displays estimates from the individual models of each domain on past-week depressive symptoms. The models progress as follows, all adjusted for the study controls: mastery (Model 1), anger suppression (Model 2), relational demands (Model 3), and all study measures (Model 4). When examined individually, having higher levels of mastery were associated with fewer depressive symptoms (Model 1; β = −0.210, *p* < 0.001) while scoring higher on anger suppression (Model 2; β = 0.481, *p* < 0.001), having no spouse (Model 3; β = 0.606, *p* < 0.001), and having some or a lot of child demands (Model 3; β = 0.406, *p* < 0.01) were significantly associated with higher depressive symptoms. As shown in Model 3, family and friend demands were unrelated to depressive symptoms. When all domains were included together (Model 4), mastery (β = −0.180, *p* < 0.001), anger suppression (β = 0.425, *p* < 0.001), no spouse (β = 0.588, *p* < 0.001), and some or a lot of child demands (β = 0.351, *p* < 0.05) remained statistically significant.

**Table 2.** Ordinary least squares regression models of mastery, anger suppression, and relational demands on depressive symptoms (CES-D, 0–8) (*N* = 1,217).

Measures	Model 1	Model 2	Model 3	Model 4
Mastery	−0.210*** (0.050)			−0.180*** (0.049)
Anger suppression		0.481*** (0.083)		0.425*** (0.083)
Spousal demands				
Some/a lot of demands			0.320 (0.198)	0.236 (0.195)
No spouse			0.606*** (0.139)	0.588*** (0.137)
Child demands				
Some/a lot of demands			0.406** (0.140)	0.351* (0.138)
No child			0.357 (0.219)	0.274 (0.216)
Family demands				
Some/a lot of demands			0.144 (0.149)	0.046 (0.147)
Friend demands				
Some/a lot of demands			−0.026 (0.203)	−0.023 (0.200)
Age	−0.044*** (0.007)	−0.040*** (0.007)	−0.045*** (0.007)	−0.040*** (0.007)
Education				
GED/high school	−0.267 (0.156)	−0.307* (0.154)	−0.259 (0.155)	−0.207 (0.153)
Some college	−0.438** (0.162)	−0.488** (0.161)	−0.467** (0.161)	−0.419** (0.159)
College graduate	−0.639*** (0.190)	−0.711*** (0.188)	−0.669*** (0.190)	−0.626*** (0.188)
Income (logged)	−0.134*** (0.036)	−0.130*** (0.036)	−0.089* (0.038)	−0.082* (0.038)
Labor force status				
Part-time	0.311 (0.187)	0.375* (0.186)	0.394* (0.187)	0.407* (0.184)
Unemployed	0.583** (0.202)	0.628** (0.201)	0.685*** (0.202)	0.638** (0.199)
Retired	0.507** (0.166)	0.557*** (0.164)	0.623*** (0.164)	0.531** (0.163)
Physical conditions (0–5+)	0.281*** (0.046)	0.300*** (0.045)	0.295*** (0.046)	0.267*** (0.045)
Constant	6.304*** (0.602)	3.986*** (0.602)	4.217*** (0.591)	3.971*** (0.671)
*R* ^2^	0.130	0.141	0.142	0.171

Source: Health and Retirement Study, 2010–2012.

Reference categories were the following: none/a little demands (spousal, child, family, and friend demands); full-time (labor force status); and less than high school (education). Standard errors in parentheses. CES-D: Center for Epidemiological Studies Depression Scale; GED: General Education Diploma.

* *p* < 0.05. **p < 0.01. ***p < 0.001.

### Clinically significant depressive symptoms

Table 3 shows the odds ratios from the individual models of each domain for clinically significant past-week depression (i.e., CES-D score of 3+). Individually, mastery was significantly associated with lower odds of clinically significant past-week depression (Model 1; OR = 0.795, *p* < 0.001) while scoring higher on anger suppression (Model 2; OR = 1.572, *p* < 0.001), having some or a lot of spousal demands (Model 3: OR = 1.795, *p* < 0.05), having no spouse (Model 3; OR = 2.285, *p* < 0.001), and having some or a lot of child demands (Model 3; OR = 1.592, *p* < 0.01) were associated with higher odds of clinically significant past-week depression. Family (Model 3) and friend (Model 3) demands were unrelated to past-week depression.

**Table 3.** Odds ratios of mastery, anger suppression, and relational demands on clinically significant depressive symptoms (3+ on CES-D) (*N* = 1,217).

Measures	Model 1	Model 2	Model 3	Model 4
Mastery	0.795*** (0.046)			0.805*** (0.048)
Anger suppression		1.527*** (0.153)		1.445*** (0.150)
Spousal demands				
Some/a lot of demands			1.795* (0.462)	1.680* (0.437)
No spouse			2.285*** (0.436)	2.268*** (0.437)
Child demands				
Some/a lot of demands			1.592** (0.265)	1.519* (0.257)
No child			1.433 (0.383)	1.355 (0.366)
Family demands				
Some/a lot of demands			1.111 (0.197)	0.997 (0.182)
Friend demands				
Some/a lot of demands			0.868 (0.209)	0.865 (0.211)
Age	0.962*** (0.008)	0.965*** (0.008)	0.961*** (0.008)	0.964*** (0.009)
Education				
GED/high school	0.743 (0.133)	0.701* (0.126)	0.745 (0.135)	0.784 (0.144)
Some college	0.600** (0.115)	0.572** (0.109)	0.582** (0.112)	0.608* (0.119)
College graduate	0.486** (0.118)	0.447*** (0.108)	0.457** (0.112)	0.472** (0.118)
Income (logged)	0.932 (0.039)	0.933 (0.038)	0.974 (0.043)	0.981 (0.044)
Labor force status				
Part-time	1.578 (0.376)	1.663* (0.399)	1.760* (0.426)	1.773* (0.434)
Unemployed	1.655* (0.404)	1.744* (0.424)	1.861* (0.460)	1.761* (0.441)
Retired	1.746** (0.361)	1.855** (0.383)	2.023*** (0.422)	1.820** (0.387)
Physical conditions (0–5+)	1.298*** (0.072)	1.333*** (0.074)	1.315*** (0.073)	1.281*** (0.073)
Constant	13.993*** (10.043)	1.470 (1.060)	1.306 (0.937)	1.403 (1.165)

Source: Health and Retirement Study, 2010–2012.

Reference categories were the following: none/a little demands (spousal, child, family, and friend demands); full-time (labor force status); and less than high school (education). Standard errors in parentheses. CES-D: Center for Epidemiological Studies Depression Scale; GED: General Education Diploma.

* *p* < 0.05. **p < 0.01. ***p < 0.001.

When all domains were included together in Model 4, mastery remained significantly associated with lower odds of clinically significant past-week depression (OR = 0.805, *p* < 0.001). Respondents had approximately a 45% increase in the odds of clinically significant past-week depression with each unit increase in anger suppression scores (OR = 1.445, *p* < 0.001). Those experiencing spousal demands were 68% more likely to experience clinically significant depression compared to those with no/little spousal demands (OR = 1.680, *p* < 0.05). Being without a spouse was associated with over twice the likelihood of having clinically significant past-week depression compared to those who had no/little spousal demands (OR = 2.268, *p* < 0.001). Black women who reported having some or a lot of child demands had 1.52 higher odds (OR = 1.519, *p* < 0.05) of clinically significant past-week depressive symptoms compared to women who had little/no child demands.

### Lifetime depression diagnosis

Table 4 shows odds ratios from lifetime diagnosed depression models. Individually, mastery (Model 1; OR = 0.800, *p* < 0.001) was significantly associated with lower odds while anger suppression (Model 2; OR = 1.768, *p* < 0.001) was associated with higher odds of lifetime diagnosed depression. Regarding relational demands, having no spouse (Model 3; OR = 1.912, *p* < 0.01) and having some/a lot of family demands (Model 3; OR = 1.487, *p* < 0.05) were associated with higher odds of lifetime diagnosed depression. Friend demands (Model 3) were not related to lifetime diagnosed depression.

**Table 4.** Odds ratios of mastery, anger suppression, and relational demands on lifetime professionally diagnosed depression (*N* = 1,217).

Measures	Model 1	Model 2	Model 3	Model 4
Mastery	0.800*** (0.051)			0.826** (0.055)
Anger suppression		1.768*** (0.197)		1.686*** (0.193)
Spousal demands				
Some/a lot of demands			1.293 (0.381)	1.159 (0.348)
No spouse			1.912** (0.404)	1.863** (0.399)
Child demands				
Some/a lot of demands			1.280 (0.238)	1.169 (0.222)
No child			1.330 (0.394)	1.232 (0.371)
Family demands				
Some/a lot of demands			1.487* (0.283)	1.332 (0.261)
Friend demands				
Some/a lot of demands			1.140 (0.289)	1.161 (0.299)
Age	0.935*** (0.009)	0.938*** (0.010)	0.936*** (0.010)	0.940*** (0.010)
Education				
GED/high school	0.854 (0.176)	0.798 (0.165)	0.852 (0.176)	0.899 (0.190)
Some college	0.967 (0.206)	0.937 (0.199)	0.932 (0.199)	0.996 (0.217)
College graduate	0.864 (0.230)	0.794 (0.213)	0.822 (0.221)	0.868 (0.240)
Income (logged)	0.921 (0.039)	0.923 (0.040)	0.961 (0.044)	0.969 (0.046)
Labor force status				
Part-time	2.618*** (0.754)	2.802*** (0.817)	2.792*** (0.812)	2.916*** (0.860)
Unemployed	3.531*** (0.987)	3.809*** (1.069)	3.857*** (1.090)	3.794*** (1.089)
Retired	4.237*** (1.049)	4.581*** (1.142)	4.744*** (1.184)	4.407*** (1.122)
Physical conditions (0–5+)	1.332*** (0.082)	1.368*** (0.084)	1.344*** (0.083)	1.315*** (0.083)
Constant	22.631*** (18.098)	1.655 (1.338)	2.324 (1.848)	1.454 (1.342)

Source: Health and Retirement Study, 2010–2012.

Reference categories were the following: none/a little demands (spousal, child, family, and friend demands); full-time (labor force status); and less than high school (education). Standard errors in parentheses. GED: General Education Diploma.

* *p* < 0.05. **p < 0.01. ***p < 0.001.

In the full model (Model 4), each unit increase in mastery was associated with an 18% decrease in the odds of being diagnosed with depression (OR = 0.826, *p* < 0.01). In addition, each unit increase in anger suppression was associated with a 69% increase in the odds of lifetime diagnosed depression (OR = 1.686, *p* < 0.001). Those without a spouse had 86% increased odds of lifetime diagnosed depression compared to those with none/a little spousal demands (OR = 1.863, *p* < 0.01).

Across our analysis, it is noteworthy that the three characteristics associated with Strong Black Womanhood emerged as significant predictors of depressive symptoms and/or depression after adjustments for more traditionally studied factors (i.e., physical health, health behaviors, and socioeconomic indicators).

## Discussion

Drawing data from a nationally representative sample of older Black women, the aim of this study was to elucidate whether three characteristics linked to Strong Black Womanhood were associated with depressive symptoms/depression: mastery, anger suppression, and relational demands (i.e., with spouse, child(ren), family, and friends). Our nuanced findings revealed that Strong Black Womanhood characteristics diminished (e.g., mastery) and augmented (e.g., anger suppression; relational demands) depression risk which reflect the complexities of traversing intersectional inequalities at the nexus of racism, sexism, and ageism for older Black women. Though the three characteristics investigated here were proxies for dimensions of the SWS framework (e.g., emotion suppression and obligation to help others), study findings lend support to Woods-Giscombé’s (2010, 2019) conceptualization of SWS endorsement yielding both assets and liabilities.

Having a sense of mastery or personal control was associated with lower risk for depression no matter how it was assessed (e.g., as a lifetime professional diagnosis or self-reported past-week symptoms). This finding is consistent with past research on older adults^33,36^ and Black women.^35,37^ Mastery may operate as a health-protective mechanism for Black women because it is tied to cultural mandates of self-sufficiency and independence. Relatedly, the health-protectiveness of mastery aligns with recent studies revealing that the SWS dimensions of either having an obligation to present strength or an intense motivation to succeed being linked to reduced anxiety symptoms,^23^ lower risk of pre-hypertension/hypertension,^67^ and higher resilience.^68^ Baker and colleagues^25^ similarly note, regarding older Black women’s endorsement of strength: “Endorsement of this cultural archetype [strong Black womanhood] may also promote resilience by providing inspiration and encouragement in increasing one’s *sense of control* and confidence when faced with obstacles” (our emphasis; p. 54). Future research should assess the role of mastery in buffering against the psychological influence of stress exposure among older Black women. If mastery emerges as an important stress buffer, self-mastery development may be an important intervention in therapeutic settings to mitigate stress among older Black women experiencing depression. This recommendation is not to minimize, however, the significant structural barriers faced by this population owing to their gendered, racialized, and age-related subordination in broader U.S. society.^69^

Consistent with research demonstrating that emotion suppression is detrimental to Black women’s mental health,^22,24^ anger suppression was associated with depression (across all three depression-related outcomes). Anger suppression may be perceived as a necessary form of comportment for Black women as they attempt to avoid stereotypical depictions of them as Sapphires or “Angry Black Women.”^54,70,71^ Socialization messages lauding “strength” as a quintessential characteristic of Black womanhood are often incompatible with expressions of anger. Moreover, given that older Black women report high levels of religiosity,^72^ their religious ideals (e.g., espousing the belief in Biblical scripture to “Be ye angry, and sin not”; Ephesians 4:26a, KJV) may be discordant with outward expressions of anger. Nevertheless, anger should not be viewed as a positive or negative emotion; instead, responses to anger may be considered adaptive or maladaptive.^39,73^ Given its association with depressive symptoms and lifetime depression, suppression appears to be a maladaptive means for processing anger. One clinical implication is that mental health care providers working with an older Black women clientele should be attuned to anger suppression as a form of culturally prescribed coping in this population, especially given the societally mandated and internalized pressure for Black women to suppress their emotions.^21,73^ In essence, addressing anger suppression among older Black women in mental health care must include cultural sensitivity to their unique racial and gender positionality.^73^ For health care providers working with older Black women, it is important to acknowledge the structural impediments to and interpersonal dynamics of anger expression. Practitioners may engage with clients about the possibility that systemic intersectional barriers over their life course could be fundamental causes of the anger older Black women feel unable to express.^27^ For interpersonal scenarios that instigate anger, mental health professionals may provide role-play opportunities and practical tools (congruent with their religious beliefs) for communicating their anger within different relationships (e.g., with an employer versus an adult child).^73^

Findings regarding relational demands revealed both anticipated and unanticipated findings. Consistent with expectation, spousal, child, and family demands were distressing for older Black women. These findings are consistent with the notion that Black women serve as self-sacrificing caregivers for their families and are related to the dimension of SWS referred to as “obligation to help others at one’s own expense.”^21,22^ Specifically, some or a lot of demands from children were associated with past-week depressive symptoms, revealing the impact of this source of demands on recent symptomatology. The dynamic child–parent relationship can be supportive, yet fraught.^74,75^ For instance, on one hand, older Black mothers (i.e., ages 51–70) are more likely to receive financial help from their adult children compared to White mothers.^76^ On the other hand, negative or ambivalent relationship quality with adult children^77^ and having adult children with stressful or problem-riddled lives^78^ are linked to higher depressive symptoms among older adults. Given the body of work demonstrating that parental stress carries over into later life for older parents who remain connected to their adult children, it is perhaps expected that demands from children would have psychological impacts on older Black mothers.

Demands from family were associated with heightened risk for lifetime professionally diagnosed depression, but not past-week depressive symptoms. Throughout their life course, obligations to family are especially high for Black women who are characterized as being the “back bone” of the Black family.^18,79^ Though the role of being a foundational component of the family is imbued with honor, it can also entail heightened responsibilities with little reciprocation. More generally, women in nonmutual relationships who endorse care as “self-sacrifice” experience heightened risk of depression because they experience a loss of self within their relationships.^50^ This phenomenon of self-sacrifice is a defining characteristic of strong Black womanhood that yields social, emotional, and economic benefits for those within an older Black woman’s social network but also unwittingly exacts a psychological toll on her.^18,21,22^

In contrast to an oversaturation of demands from children and family members, a sizable portion of Black women (nearly two-thirds) did not have a spouse and those without a spouse were particularly vulnerable to higher depressive symptoms and lifetime depression. We conducted supplemental analysis distinguishing between divorced/separated, widowed, and never-married Black women, and found that each status was associated with higher depressive symptoms and lifetime depression compared to respondents with few or no demands from their spouse. However, separated/divorced and never-married older Black women had higher risk of depressive symptoms than those who were widowed. Regarding lifetime depression, separated/divorced and widowed respondents had higher risk of lifetime depression than their never-married counterparts. These findings are likely attributable to both a loss in emotional and financial support for those who were widowed or divorced/separated,^80^ and failure to meet societally imposed expectations of marriage for those who were never married. Not only are Black women expected to be strong and supportive of everyone in the community, but they are also expected to do so as devoted wives.^17,18^ Singlehood, particularly never marrying, is viewed in broader U.S. culture and among Black communities as a deficiency or personal failure.^81,82^ This cohort of Black women, born in 1960 or earlier, were socialized during a time when marriage was expected and the “respectable” thing to do, thereby placing a high premium on marriage.^79,83^ In essence, both external and internal expectations of marriage may impose psychological harm on never-married Black women in this age group.

Though marriage is a less common status among older Black women, the 12% of respondents who reported some or a lot of spousal demands were at greater risk of clinically significant past-week depressive symptoms compared to their counterparts with little or no spousal demands. Our findings underscore conclusions drawn from the broader literature which indicate that marriage alone is not mental health protective.^84,85^ Instead, marital quality—with spousal demands being one indicator of marital quality—appears more consequential than simply the status of being married. Thus, our findings align with Hanus and colleagues’ recent study focused on mid- and later-life Black women.^9^

Despite our expectation that Black women’s depressive symptoms would be impacted by demands across all relationships, friend-involved demands never emerged as a significant predictor of depression. Perhaps because friendships are voluntary relationships, it is relatively easier to exit them (compared to children or relatives), thereby not significantly contributing to depression in this population. When caring for family, children, or a spouse, demands may be perceived as obligatory and inescapable. However, responding to friends’ demands, though there may be expectations of reciprocity, is a voluntary act. Relatedly, friendships may be more reciprocal and less imbalanced compared to familial relational dynamics. Older Black women may find caring for and supporting friends enjoyable, particularly close relationships with other Black women.^86,87^ Research on sister circles also indicates that Black women provide one another support that encourages vulnerability over “strength,” and privileges self-care over self-sacrifice.^88^ In essence, demands from friends may be unrelated to depression among older Black women because friends provide a haven of mutual understanding and communal belonging.

Taken together, our findings demonstrate the utility of integrating intersectionality, the Strong Black woman construct, and the SWS framework to identify specific, culturally relevant factors associated with depression risk among older Black women. It is important to recognize the historical and social embeddedness of the Strong Black woman archetype and its necessity for Black women’s survival. The intersectional and contextual processes underlying our study findings provide insights on how older Black women have survived despite facing overlapping systems of inequality. For instance, the cohort of older Black women studied here may have been socialized to suppress anger during a period (i.e., under the Jim Crow regime) in which outward anger expression could have elicited a violent response. On the other hand, under the weight of explicit racism and sexism, developing a sense of mastery provided the confidence needed to strategically maneuver through systems not designed for Black women’s success (e.g., institutions of higher education). In sum, the Strong Black Woman archetype provides a critical sociohistorical lens through which to understand the psychosocial determinants of depression among older Black women.

### Limitations

Despite study strengths, our findings must also be interpreted in the context of important limitations. First, though we assessed three measures of depression and depressive symptomatology, there is a possibility that our estimates are undercounts. Because of the pervasive nature of the Strong Black Woman ideal, some respondents may have been unwilling to report feeling symptoms associated with depression. An admission of depressive symptoms could be incompatible with conceptualizations of strength among some Black women.^59^ Regarding lifetime diagnosis of depression, given that Black women are less likely to engage in mental health care help-seeking compared to other groups, there is possibility that the prevalence of diagnosed depression is higher than the 20% who reported being professionally diagnosed with depression. This is particularly relevant for older Black women who may feel more comfortable seeking guidance from religious leaders and clergy as opposed to mental health care professionals.^89,90^ Second, the quantitative scale developed to assess SWS was not available in the data. Thus, a more direct test of the relationship between SWS and depression among older Black women awaits future empirical study. Nevertheless, a key strength of the data used for this analysis is that it is a nationally representative sample, which allows for potential generalizability of our study findings to older U.S. Black women. Third, Black women are a diverse group comprised of individuals from various ethnic and cultural backgrounds. For instance, recent estimates indicate that approximately 21% of Black Americans are either immigrants or the children of immigrants.^91^ In our analytic sample, 5% (*N* = 64) of respondents were foreign-born, preventing us from investigating how nativity status might play a role in the factors examined in this study. Future research focused on strong Black womanhood should investigate how these social processes affect depression risk among older Black immigrant women.

## Conclusion

Strong Black Womanhood, though offering an ostensibly positive depiction of Black women, entails characteristics that have both positive and negative mental health impacts. Our results, on one hand, showed that older Black women with a high sense of mastery experienced fewer depressive symptoms and lower risk of lifetime depression. On the other hand, anger suppression was linked to higher depressive symptoms and lifetime depression. Demands from one’s spouse, children, and family members emerged as significant predictors of either depressive symptoms or lifetime depression. Moreover, unmarried older Black women are at heightened risk for depression. Mental health care providers should be aware of these specific psychosocial correlates of depression among older Black women and use this to inform their assessments and care for this population.

## Note

Various terms are used to capture the notion of the “Strong Black Woman” across different fields of study. For instance, “Sojourner Truth Syndrome,” “Sisterella Complex,” and “Strong Black Woman Schema” are concepts used to describe aspects of the Strong Black woman ideal.^92–97^ Here, we draw inspiration from the SWS framework because it details five distinct characteristics linked to the broader overarching concept of “Strong Black Womanhood.” However, we would be remiss not to mention other scholars who developed quantitative measures of Strong Black Womanhood. For example, the Stereotypic Roles for Black Women Scale (SRBWS) includes four dimensions: Mammy, Sapphire, Jezebel, and Superwoman.^97^ The African American Women’s Shifting Scale (AAWSS) also includes a “Strong Black Woman” subscale.^95^ Nevertheless, the measures available in the HRS most clearly align with the SWS framework developed by Woods-Giscombé.^21–22^

## Declarations

**Ethics approval and consent to participate:** Collection of Health and Retirement Study data was approved by the Institutional Review Board at the University of Michigan, the location at which the data are housed (approval number: HUM00061128). Per the HRS website, “Prior to each interview, participants are provided with a written informed consent information document. At the start of each interview, all respondents are read a confidentiality statement and give oral consent by agreeing to do the interview” (https://hrs.isr.umich.edu/sites/default/files/biblio/HRS_IRB_Information%28web%29_08_2018.pdf).

**Consent for publication:** Implied consent for publication was taken when respondents agreed to complete the questionnaire administered in the study.

**Availability of data and materials:** Health and Retirement Study data are available for public use at the following website: https://hrs.isr.umich.edu/data-products
